# *Long-Term Follow-Up* of Tufting Enteropathy Caused by *EPCAM* Mutation p.Asp253Asn and Absent EPCAM Expression

**DOI:** 10.1097/PG9.0000000000000029

**Published:** 2020-12-03

**Authors:** Oğuz Ozler, Andrea Brunner-Véber, Parmis Fatih, Thomas Müller, Andreas R. Janecke, Cigdem Arikan

**Affiliations:** From the * Koc University School of Medicine, Pediatric Gastroenterology and Hepatology, Organ Transplantation and Research Center, Koc University Research Center for Translational Medicine (KUTTAM), Istanbul, Turkey; † Institut für Pathologie, Neuropathologie und Molekularpathologie, Medical University of Innsbruck, Innsbruck, Austria; ‡ Department of Pediatrics I, Medical University of Innsbruck, Innsbruck, Austria; § Division of Human Genetics, Medical University of Innsbruck, Innsbruck, Austria.

**Keywords:** arthritis, catch-up growth, favorable outcome, parenteral nutrition, weaning

## Abstract

Tufting enteropathy (TE) is caused by recessive epithelial cell adhesion molecule (*EPCAM*) mutations, features congenital intractable diarrhea, growth retardation, and a characteristic disorganization of surface enterocytes. TE generally requires parenteral nutrition (PN) throughout childhood and into adulthood or a small bowel transplantation. We report 2 siblings with TE; a 3-year-old patient 1 intermittently receives partial PN, monthly somatostatin therapy, tolerates a normal diet and has a normal stool output. However, she is the sixth patient of 90 TE patients in literature, to develop a chronic arthritis. A 12-year-old patient 2 is on a normal diet, and did not require PN for the past 8 years. Duodenal biopsies showed characteristic tufts, and a complete lack of EPCAM staining. Both siblings were homozygous for EPCAM mutation c.757G>A (p.Asp253Asn). This observation shows that an overall favorable outcome can be obtained in TE, even with abrogated intestinal EPCAM expression.

**What Is Known**

- Tufting enteropathy (TE) features congenital diarrhea, growth retardation and a characteristic disorganization of surface enterocytes.
- TE most often requires parenteral nutrition (PN) throughout childhood, but weaning from PN and a favorable outcome was reported.
- TE is caused by *EPCAM* mutations, many of which abrogate EPCAM production.

**What Is New**

- We report 2 siblings with TE who tolerate a normal diet at 3 and 12 years of age, are homozygous for EPCAM mutation p.Asp253Asn, and lack intestinal EPCAM expression.
- A favorable outcome in TE does not depend on residual EPCAM expression.
- Arthritis is reported in 6 of ~90 patients reported with TE.

**T**ufting enteropathy (TE) is a rare inherited enteropathy (OMIM #613217) presenting with intractable watery diarrhea (ID) and impaired growth in infancy. Histologically, it is characterized by disorganization of surface epithelium with focal crowding, resembling tufts, villous atrophy without mononuclear cell infiltration, and basement membrane abnormalities. TE has a wide range of severity, but patients generally require parenteral nutrition (PN) for several years or throughout childhood (1–3). Prolonged PN therapy leads to complications such as sepsis, thrombosis, liver disease, and poor quality of life. In the complicated cases, small bowel transplant is a therapeutic option. However, a 3-year survival rate after transplant is around 30% (4).

Its prevalence is estimated with 1/50,000–100,000 live births in Western Europe and is higher in countries with frequent consanguineous marriages. Mutations in the gene encoding human epithelial cell adhesion molecule (EPCAM) were first identified in TE in 2008 (4), while mutations in SPINT2 account for patients with syndromic TE (5, 6). In this study, we report the long-term outcome and the histopathology in 2 Turkish siblings with TE and the EPCAM mutation p.Asp253Asn, which shows the outcome does not depend on residual EPCAM expression.

## CASE REPORTS

### Patient 1

**FIGURE 1.**
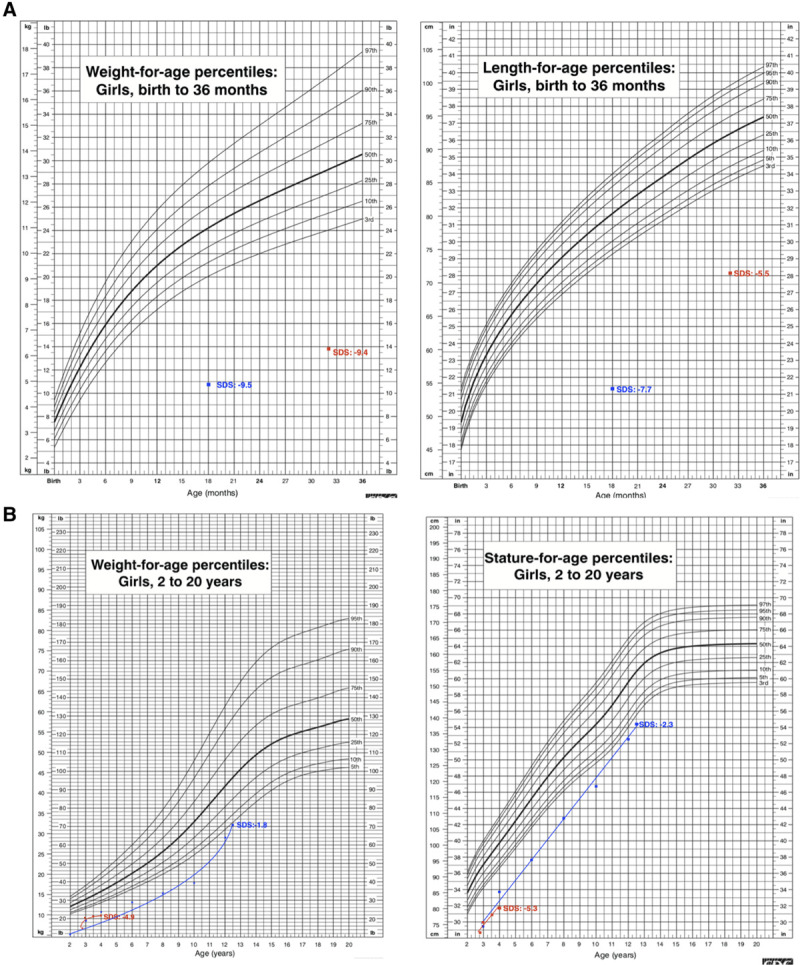
Growth parameters in patient 1 (red line) and patient 2 (blue line) were showed significant improvement during follow-up.

A 3-year old female suffering from ID since she was 13 days old presented to our clinic with chronic malnutrition and dehydration. She displayed vomiting and bulky diarrhea with 2000–2500 g of stool output each day. Duodenal biopsy results showed a disorganization of epithelial enterocytes and villus tufts. Her diarrheal attacks had already caused frequent hospitalizations for rehydration and to treat severe metabolic acidosis. She was born after an uneventful pregnancy by cesarean section as the second living child to consanguineous parents (first cousins). Her mother was healthy; her sister had a history of ID in childhood, but no particular diagnosis was made. Her birth weight and height were 3200 g and 51 cm, respectively. On admission, the patient was dehydrated and pale, with tachypnea and a distended abdomen. There were no dysmorphic features. Her weight and height were 6300 g and 71.5 cm, respectively, (SDS –9.4 and –5.5, respectively, Fig. 1A). Stool investigations for infectious agents was negative but electrolytes suggested a mixed osmotic and secretory type of diarrhea: sodium (10 mEq/L), chloride (4 mEq/L), pH (7.0), and osmotic gap (257 mOsm/kg) (23.18 mg/dL). Routine testing showed mild microcytic anemia and hypophosphatemia, and was otherwise normal. She received fluid and electrolyte replacement targeting acidosis and dehydration via TPN. Octreotide infusion of 1 mcg/kg/min was started concomitantly. Enteral nutrition (EN) was introduced after the stool output normalized, and diet was oriented on her vomiting and stool changes. She received home TPN for 2 months, and continues with monthly intramuscular somatostatin therapy, a normal enteral diet and normal stool output. She shows catch-up growth under hydrolyzed formula, with weight and height at age 4 years are 9.8 kg (–4.9 SDS) and 80 cm (–5.3 SDS) (Fig. 1A).

Within the last 6 months, she experienced 6 episodes of painful swelling of her hands, without fever, rash, or stool changes. Ibuprofen was initiated, and her symptoms resolved within 1 week each time. Laboratory investigations were unremarkable in terms of autoimmune markers, but CRP, serum amyloid A, and thrombocyte counts were elevated. Targeted genetic testing excluded concomitant Familial Mediterranean Fever.

### Patient 2

This is the 12-year-old sister of the first patient. She first presented to our clinic aged 18 months with ID, and failure to thrive (weight and height SDS –9.5 and –7.7, respectively, Fig. 1B). Duodenal biopsy results showed a disorganization of epithelial enterocytes and villus tufts. Her diarrheal attacks had led to hospitalizations elsewhere for severe dehydration and metabolic acidosis. As in her sister, her laboratory studies revealed osmotic and secretory type of diarrhea and could only be controlled with stopping EN. Somatostatin was used as supportive treatment. She was initially TPN-dependent, but was weaned gradually, PPN was stopped at age 4 years, and she shows catch-up growth since then. An episode of catheter infection and thrombosis had been treated successfully. Her weight and height are now 32 kg (–1.8 SDS) and 138 cm (–2.3 SDS); her ID completely resolved and she can consume everything and participates in daily life.

## MOLECULAR INVESTIGATION AND RESULTS

Written informed consent for diagnostic molecular genetic testing was obtained from the parents of the patients. A homozygous *EPCAM* missense mutation c.757G>A (p.Asp253Asn) was identified in case 2 by WES (7), and it segregated with disease in the family. The mutation localizes within a 29-Mb region of homozygosity on chromosome 2p21 in patient 2, in line with the hypothesis of identity-by-descent of the disease-causing mutation due to parental consanguinity. This missense mutation is predicted to be damaging by all 3 in silico programs and it is not listed in the ExAC and gnomAD databases, indicating that it is not a frequent polymorphism.

**FIGURE 2.**
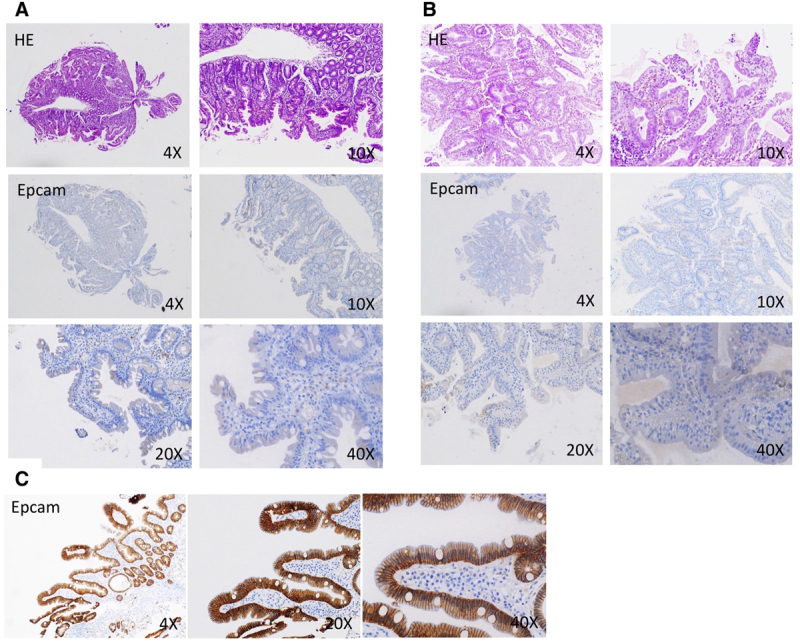
Lack of EPCAM protein expression caused by the p.Asp253Asn mutation. There was no EPCAM staining detected in FFPE slides of duodenal biopsies from patients 1 (A; H&E, magnification 4× and 20×; EPCAM immunohistochemistry, magnification 4×, 10×, 20×, and 40×) and patient 2 (B; H&E, magnification 4× and 20×; EPCAM immunohistochemistry, magnification 4×, 10×, 20×, and 40×), as compared to an on-slight control (C; EPCAM immunohistochemistry, magnification 4×, 20×, and 40×) using a monoclonal EPCAM antibody (Clone Ber-EP4, Cell Marque, Millipore Sigma). EPCAM, epithelial cell adhesion molecule.

Duodenal biopsies demonstrated partial villous atrophy, crypt hyperplasia, and normal amounts of intraepithelial lymphocytes in both patients. Surface epithelium displayed disorganization of enterocytes with focal tufting, and complete lack of EPCAM staining on immunohistochemistry (Fig. 2A, B) as compared to on-slide control (Fig. 2C).

## DISCUSSION

EPCAM is a protein expressed at the basolateral membrane of intestinal epithelial cells mainly functioning in cell–cell interactions through homooligomerization. It is connected to the actin cytoskeleton and interacts with claudin-7 to modulate tight junctions. In TE, this interaction is lost causing intestinal barrier disruption (8). EPCAM mutations were identified as responsible for TE in 90 reported cases (9). TE patients who do not harbor EPCAM mutations generally present the syndromic form of TE, caused by mutations in *SPINT2* (MIM# 605124) (2). Here, we present 2 Turkish siblings with TE and a homozygous c.757G>A (p.Asp253Asn) mutation in the EPCAM gene. This mutation was reported in a single patient of Turkish origin who was noted to require total PN, but other clinical information was not provided (9). This previous report and our observations support the hypothesis that the p.Asp253Asn mutation underlies TE in these patients. Most importantly, our demonstrating negative EPCAM immunohistochemistry in both patients’ intestinal biopsies provides final evidence for this mutation’s pathogenicity. The mutated aspartate-253 is buried within the EpCAM molecule (9), and the mutation most likely causes protein misfolding and degradation.

We provide one of the rare long-term follow-up reports of TE. Frequent hospital admissions were necessary to treat severe acidosis and dehydration in the first years of life in our patients, and TPN and octreotide treatment were provided at these occasions. The severity of the diarrhea decreased after the age of 4 years. Both patients gradually tolerated increased EN. However, both patients displayed severely stunted growth at the first admission. Patient 2 is in complete remission for 8 years, without any diarrheal symptoms, and shows catch-up growth. A disease course like that in our patients is infrequently reported among TE patients with EPCAM mutations; in part, this is due to the rarity of this condition. They generally require TPN or PPN from early infancy on and are TPN or PPN-dependent at the time of their reporting. Of the 90 reported patients, 13 patients underwent intestinal transplant and 12 patients were deceased, highlighting the marked morbidity and mortality of this condition (9).

Our report shows that patients with EPCAM-related TE, who receive PN in their early years of most pronounced body growth, and in whom PN-related complications are addressed can be weaned from PN and achieve normal growth and psychomotor development. In addition, intraluminal nutrients have stimulatory effects on epithelial cells and on trophic hormones that enhance intestinal adaptation and reduce the need for parenteral support. Hydrolyzed proteins seem to confer a significant absorptive advantage over amino acid preparations alone and seem to be more trophic to the gut than the intact proteins as reported previously. Favorable outcome at long-term follow-up has been reported in 2 case-series, but EPCAM mutation analysis and immunohistochemistry were not performed in one study (3), and protein expression was not studied in the other study (10).

The small number of genetically characterized TE patients, with a large number of different EPCAM genotypes, hinders a genotype-phenotype correlation. However, as a complete lack of intestinal EPCAM protein was revealed in our patients, which represent a common histopathologic finding among patients with TE and is particularly true for patients with homozygous nonsense mutations (2, 4), we are tempted to speculate that weaning from PN might depend on factors other than the EPCAM genotype in such patients.

Importantly, we observed tenosynovitis in patient one. Five such patients have been reported reinforcing the idea patients with TE are prone to develop chronic arthritis (10–12), albeit its pathophysiology is elusive. EPCAM has multiple functions both under physiologic and pathologic conditions due to its expression in many types of epithelia in humans. The intestinal barrier function is impaired in EPCAM knockout mice, which may induce adaptive immunity as a consequence to protect the intestines from inflammation. EPCAM may also protect intestines from inflammation via binding extra vesicles containing TGF-β1 (13).

In conclusion, severe diarrhea and growth failure due to TE should be followed closely before transplantation decision and somatostatin and hydrolyzed formula may decrease PN-requirements and avoid BT in the long term.
